# Tafasitamab in refractory diffuse large B-cell lymphoma with neurolymphomatosis

**DOI:** 10.1007/s00277-025-06184-6

**Published:** 2025-02-12

**Authors:** João Ricardo Belo Freitas Mendes, Laurine Couleur, Chloe Manca, Valérie Frossard, Mitja Nabergoj

**Affiliations:** 1 Department of Internal Medicine, Hôpital Riviera-Chablais Vaud-Valais, Rennaz, Switzerland; 2 Division of Hematology and Laboratory of Hematology, Institut Central des Hôpitaux, Sion, Switzerland; 3 Nuclear Medicine, Hôpital Riviera-Chablais Vaud-Valais, Rennaz, Switzerland

**Keywords:** DLBCL, Neurolymphomatosis, Tafasitamab, Lenalidomide

## Abstract

Peripheral nervous system involvement in lymphoproliferative diseases, often due to direct nerve infiltration (neurolymphomatosis, NL), is mostly seen in aggressive B-cell lymphoma. We report the case of an 88-year-old man with stage IVA DLBCL, who achieved the first complete response after six R-miniCHOP21 cycles. One year post-treatment, he developed severe neurological symptoms, and PET-CT revealed widespread relapse with extensive neural involvement. Treatment with tafasitamab and lenalidomide led to a complete morpho-metabolic remission and full neurological recovery, with minimal side effects. This case underscores for the very first time the efficacy and tolerability of this regimen in treating NL, highlighting its potential for frail patients unfit for more intensive therapies.

Peripheral nervous system involvement can occur at any stage of lymphoproliferative diseases due to various mechanisms, frequently from direct nerve infiltration (neurolymphomatosis) [1]. Most often, neurolymphomatosis (NL) occurs in aggressive B-cell lymphoma [2]. A high index of suspicion, neurophysiological studies and biopsy are needed to confirm neural involvement. Imaging techniques like MRI and PET can replace this invasive procedure [3, 4]. PET-CT has proven to be both specific and sensitive in identifying neural involvement in NL [5]. Treatment options include immunochemotherapy regimens (e.g. R-CHOP), high-dose methotrexate and radiotherapy. In the context of relapsed/refractory diffuse large B-cell lymphoma (R/R DLBCL) with NL, the prognosis is dire [6]. More aggressive treatments, including autologous stem cell transplantation, may offer a survival advantage [7]. Emerging therapies like CAR-T cell therapy show promise but may carry an increased risk of toxicity.

Tafasitamab in combination with lenalidomide has shown durable responses in combinations with lenalidomide in patients with R/R DLBCL who are not eligible for ASCT [8].

We present a case of an 88-year-old healthy man with stage IVA diffuse large B-cell lymphoma (DLBCL), NOS, of ABC molecular subtype. After six R-miniCHOP21 cycles, the post-treatment PET-CT showed a complete morpho-metabolic response.

One year after the completion treatment, the patient complained following a fall of progressive left upper limb weakness, leading progressively to complete arm palsy and muscle atrophy (Erb’s palsy). EMG showed severe denervation, suggesting post-traumatic plexus damage. A month later, a progressive reappearance of left cervical adenopathies was noticed. PET-CT revealed a widespread nodal and extranodal involvement, with pericardiac, pleural, renal, pancreatic, gastric, subcutaneous and muscular lesions. A large mass involving the 6th cervical vertebral body, extending to the left foraminal and spinal canal, and the left brachial plexus and nerves was observed (Fig. 1A).

**Fig. 1:**
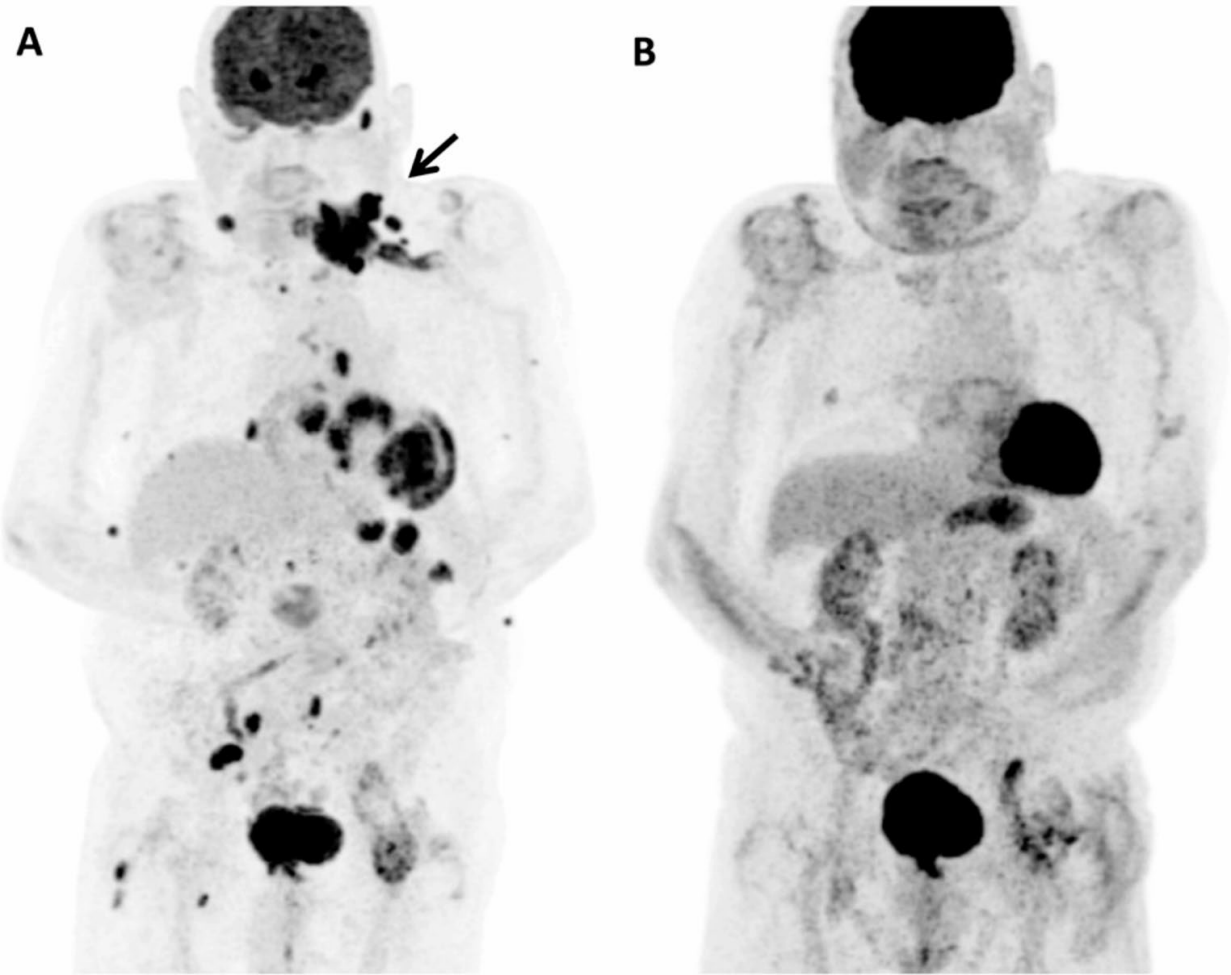
(**A**) 18 F-FDG PET MIP at relapse showing nodal and extra-nodal involvement at relapse. Bold arrow indicates lymph node cluster in the left supraclavicular region with intense hypermetabolism (SUV max 21.1) involving the 6th cervical vertebral body with extension to the left foraminal and spinal canal, left brachial plexus and nerves. (**B**) 18 F-FDG PET MIP after C9 showing a complete metabolic response

Given the late relapse (> 12 months after previous treatment), and reduced performance status, therapy with tafasitamab and lenalidomide was initiated with reduced lenalidomide dosing (15 mg/d, adapted to renal clearance). After two cycles, PET-CT showed partial remission. PET-CT after nine cycles eventually confirmed complete response (Fig. 1B). Progressive neurological improvement was observed from third treatment cycle, with full neurological recovery after one year of treatment start. Treatment tolerance was good: patient suffered from moderate SARS-CoV2 infection during cycle 1 and a mild rash occurred during cycles 5 and 7, probably due to lenalidomide, which resolved with temporary discontinuation and steroids. The patient completed 16 cycles of treatment, and afterwards discontinued treatment at his request. A PET-CT three months later confirmed persistent complete response.

We present a case of an unfit and elderly patient with widespread relapse and secondary neurolymphomatosis. Treatment with tafasitamab and lenalidomide resulted in complete morpho-metabolic remission and full neurological recovery, with no major side effects. The efficacy of this regimen had been previously proven in cases of refractory CNS lymphomas [9]. This case highlights the efficacy and excellent tolerability of tafasitamab and lenalidomide in neurolymphomatosis and represents the first report of their effectiveness in this setting, underscoring its significant therapeutic potential for treating lymphoma with nervous system involvement in very frail patients not otherwise fit for a more intensive approach.

## Data Availability

No datasets were generated or analysed during the current study.

## Declarations

### Patient

Informed written consent was obtained from the patient for publication.
